# Efficacy and Safety of Oral Beclomethasone Dipropionate in Ulcerative Colitis: A Systematic Review and Meta-Analysis

**DOI:** 10.1371/journal.pone.0166455

**Published:** 2016-11-15

**Authors:** Francesco Manguso, Raffaele Bennato, Giovanni Lombardi, Elisabetta Riccio, Giuseppe Costantino, Walter Fries

**Affiliations:** 1 Department of Transplantation, UOSC of Gastroenterology and Endoscopy, AORN ‘A. Cardarelli’, Napoli, Italy; 2 Department of Clinical and Experimental Medicine, Clinical Unit for Chronic Bowel Disorders, University of Messina, Messina, Italy; University Hospital Llandough, UNITED KINGDOM

## Abstract

**Background and Aim:**

We performed a systematic review and meta-analysis of all the available evidence comparing efficacy and safety of oral prolonged released beclomethasone dipropionate (BDP) to active oral controls in patients with mild-to-moderate ulcerative colitis (UC). A subgroup-analysis compared the effectiveness of BDP and 5-ASA.

**Methods:**

Literature research was performed in different databases, as well as manual search to identify abstracts from international meetings with data not included in extensive publications. Experts in the field and companies involved in BDP development and manufacture were contacted to identify unpublished studies used for registration purposes. Dichotomous data were pooled to obtain odds ratio meta-analysis.

**Results:**

Five randomized controlled trials that compared oral BDP 5mg/day vs. all oral active controls in treating UC were identified as eligible. Efficacy and safety have been addressed after 4-week treatment period. One study evaluated efficacy and safety of BDP vs. prednisone and 4 of BDP vs. 5-ASA. Treatment with oral BDP 5 mg/day induces a significant better clinical response compared to oral 5-ASA (OR 1.86, 95% CI = 1.23–2.82, P = 0.003). The effect is detectable even when the comparison to prednisone is added (OR 1.41, 95% CI = 1.03–1.93, P = 0.03). Data on remission indicate that the potential clinical efficacy of BDP may be better than 5-ASA (OR 1.55, 95% CI = 1.00–2.40, P = 0.05). This difference is lost when the comparison with prednisone is added (OR 1.30, 95% CI = 0.76–2.23, P = 0.34). The safety analysis showed no differences between BDP and 5-ASA (OR 0.55, 95% CI = 0.24–1.27, P = 0.16). The lack of difference is maintained even when the study with prednisone is added (OR 0.67, 95% CI = 0.44–1.01, P = 0.06). However, the trend of difference is clear and indicates a more favourable safety profile of BDP compared to 5-ASA and PD.

**Conclusions:**

Oral prolonged release BDP showed a superior efficacy vs. oral 5-ASA in inducing clinical improvement of mild-to-moderate UC with a similar safety profile.

## Introduction

Ulcerative colitis (UC) is a chronic idiopathic inflammatory disease of the large bowel that always involves the distal portion and extends in a continuous fashion for a variable length proximally. It primarily affects late adolescence or early adult life, with a small peak in incidence demonstrated in some populations after the fifth decade of life. The majority of patients suffer from recurrent attacks at irregular intervals. The more frequent symptoms are diarrhoea, which is usually bloody, mucus with stools and abdominal pain. Type of symptoms tends to differ according to the extent and severity of the disease [1], and the most frequent active forms of UC are of mild or moderate intensity [2]. The treatment of UC has changed over the last decades. To date, the conventional therapeutic approach is based on 5-aminosalicylic acid (5-ASA), corticosteroids (CSs), immunosuppressants, and, more recently, on anti-TNF and non-anti-TNF biologic agents, all with the purpose of inducing and maintaining remission [3]. It has been shown that oral and topical CSs improve the chances of obtaining a rapid remission whatever the grade of severity of the attack, based on the anti-inflammatory property and on immunomodulation. Unfortunately, the efficacy of CSs is accompanied by a marked increase in adverse events (AEs) related to systemic absorption and to the inhibition of the hypothalamic-pituitary-adrenal (HPA) axis [4–6]. To avoid steroid associated side effects, a ‘second generation’ of topically acting steroids, with minimal systemic activity mainly because of extensive first pass liver metabolism following absorption from the gastrointestinal tract was developed [7].

Beclomethasone dipropionate (BDP), a second-generation steroid with minimal systemic activity, was synthesized by Glaxo in the 1960s and developed by Allen & Hanbury’s Ltd. (a subsidiary of the Glaxo Group) [8,9]. It was first used as a topical ointment for eczema and afterward in aerosol form for the treatment of allergic asthma [10]. Its use in inflammatory bowel disease (IBD) started in the early 1980s [11]. Topically acting retention enemas, foams or suppositories of BDP have been used successfully to treat patients with distal UC [12–22]. A first attempt to develop an oral controlled-release formulation of BDP that would enhance delivery of a pharmacologically active drug to the terminal ileum and right colon was made in 1986 for patients with IBD [23]. More recently, oral prolonged release preparation of BDP with an acid-resistant film-coating (Eudragit-L100/55) that dissolves at pH<6, releasing the active drug in the distal small bowel and colon, has been used in more extensive UC [24–33] and other gastrointestinal diseases such as pouchitis [34], Crohn’s disease [35], gastrointestinal graft-versus-host disease [36], lymphocytic colitis [37], and segmental colitis associated with diverticulosis [38]. When used in low doses, BDP has been shown to be free of many of the deleterious side effects associated with systemically absorbed CSs [23]. This is undoubtedly related to the surface-active anti-inflammatory properties of BDP, which have been reported 5000 times more potent topically than hydrocortisone [7] and 500 times than dexamethasone [39], as measured by vasoconstriction assay.

Oral BDP for UC has been approved in a number of European and non-European countries [1], but only a few randomized controlled trials (RCTs) have been produced to evaluate the efficacy and safety of oral BDP in UC patients [24–25,28,30–33]. Given that few data have been published in the field, we conducted a meta-analysis to estimate the efficacy and safety of oral prolonged release of BDP 5 mg/day vs. all oral active controls in adult patients with extensive or left-sided mild to moderate UC.

## Materials and Methods

### Search strategy and study selection

In compliance with PRISMA guidelines [40] we performed a medical literature search in PubMed (1946 to Sept 2016), EMBASE (1947 to Sept 2016), OVID, Scopus, ScienceDirect, the Cochrane central register of controlled trials, and in databases of ISI Web of Science. Moreover, abstract books of conference proceedings from the British Society of Gastroenterology, Digestive Diseases Week, United European Gastroenterology Week, European Crohn’s and Colitis Organization, and from other relevant international meetings with data not included in extenso publications were hand-searched to identify other potentially eligible studies. The bibliographies of all identified relevant studies were used to perform a recursive search of the literature. In addition, experts in the field and companies involved in the development and manufacture of BDP were also contacted to try to identify other unpublished studies used for registration purposes. We searched for RCTs comparing BDP (5 mg/day) and all active controls orally administered as tablets for at least 4-week duration, including adult patients with mild to moderate UC. The extension of the disease was left-sided or extensive colitis.

To establish a partial or complete clinical remission (response) of the intestinal disease we considered those studies in which Disease Activity Index (DAI) [41] or Clinical Activity Index (CAI) [42] was used. DAI is a 12-point scoring system that includes clinical (stool frequency, rectal bleeding, and physician’s assessment of disease activity) and endoscopic (mucosal appearance) parameters for the evaluation of the disease. It is based on the modification of Truelove’s criteria [43] for classifying the degree of clinical activity and Baron’s criteria [44] for grading the endoscopic appearance. The physician’s global assessment of disease activity (normal, mild, moderate, and severe) reflects symptoms recorded by the patient, endoscopic findings, and clinical indices. CAI score is based on clinical signs and symptoms of UC, and the total index score ranges from 0 to 29 points. A score of more than 12 denotes severe UC, from 5 to 12 mild to moderate activity, and 4 or less inactive disease. The Truelove and Richard’s grading system [45] was allowed when histological specimens were graded for the degree of inflammation.

The literature research used the following terms: ulcerative colitis, inflammatory bowel disease, colitis (both as medical subject headings and free text terms). These were combined using the set operator AND with studies identified with Beclomethasone dipropionate, BDP, or Clipper (commercial name) as free text terms. There were no language restrictions. Abstracts of the papers identified by the initial search were evaluated by the lead investigator for appropriateness to the study question, and all potentially relevant papers were obtained and evaluated in detail. Foreign language papers were translated where necessary. Articles were assessed independently by two investigators using pre-designed eligibility forms, according to the pre-defined eligibility criteria. Any disagreement between investigators was resolved by discussion.

### Outcome assessment

All outcome measures were predefined as binary variables. The first outcome was the proportion of patients reporting improvement or resolution of symptoms after any treatment they received (oral BDP 5 mg vs. all oral active controls). Oral active controls were represented by prednisone (PD) and 5-ASA. A subgroup-analysis compared the effectiveness of oral BDP vs. oral 5-ASA alone. For this purpose, we used the criteria for defining a ‘responder’ or a patient in ‘remission’ chosen by the authors of each paper, as these differed slightly across studies. Moreover, a second outcome measure was the proportion of patients suffering from drug-related AEs as reported by authors. An analysis of treatment effect was performed on intention-to-treat (ITT) basis considering dropouts and missing data as treatment failures.

### Data extraction

Data were extracted independently by two investigators (FM and WF) on a Microsoft Excel 2016 spreadsheet running on Windows 10 Pro 64-bit version (Microsoft Corp, Redmond, WA, USA). Other data were extracted for each trial, where available: country of origin, disease extension, dose used of oral BDP and of other active controls, dose allowed of oral 5-ASA compounds, follow-up duration. Data were extracted as ITT, wherever trial reporting allowed this. The data extracted were compared for any difference. If evidenced, differences were resolved by discussion and consensus between researchers.

### Assessment of risk bias

The risks of bias for RCT studies were evaluated with the Cochrane Collaboration’s Risk of Bias Tool. We assessed selection bias (random sequence generation and allocation concealment), performance bias (blinding of the study personnel as to which intervention an UC patient had received), detection bias (blinding of personnel evaluating outcomes), attrition bias (completeness of reporting data, reason and balance across groups of missing data), reporting bias (reporting of the prespecified or expected study outcomes of interest to the review) and other source of bias (early interruption of the trial due to data-dependent process or bias related to the specific study design). For each study we categorised the risks of bias as high, low or unclear, using standard methods [46]. The risk of bias was assessed independently by two researchers using a specific form. Differences in opinion were resolved by discussion and consensus. The corresponding authors of selected studies were contacted when information useful to assess the risks of bias was unclear or missing in their manuscripts as published.

### Data synthesis and statistical analysis

A meta-analysis was conducted using the software Review Manager 5.3 (Cochrane Collaboration, http://tech.cochrane.org/revman/download). For dichotomous outcomes, the odds ratio (OR) and 95% confidence interval (CI) were calculated. Raw data for each outcome were extracted and converted into individual 2×2tables. The Cochrane Handbook's Q test and I^2^ statistic were used to determine the heterogeneity among the studies [47]. If there was significant heterogeneity (P <0.05 or I^2^ > 50%), a random-effects model was used. Otherwise, fixed-effects models were applied if there was no significant heterogeneity (P ≥ 0.05 and I^2^ ≤ 50%). In this meta-analysis, if the degree of heterogeneity was greater than the statistical test results, a sensitivity analysis was performed. Funnel plots were generated to assess the publication bias. P values were 2 tailed, and the statistical significance was set at 0.05.

## Results

### Studies’ characteristics

**Fig 1:**
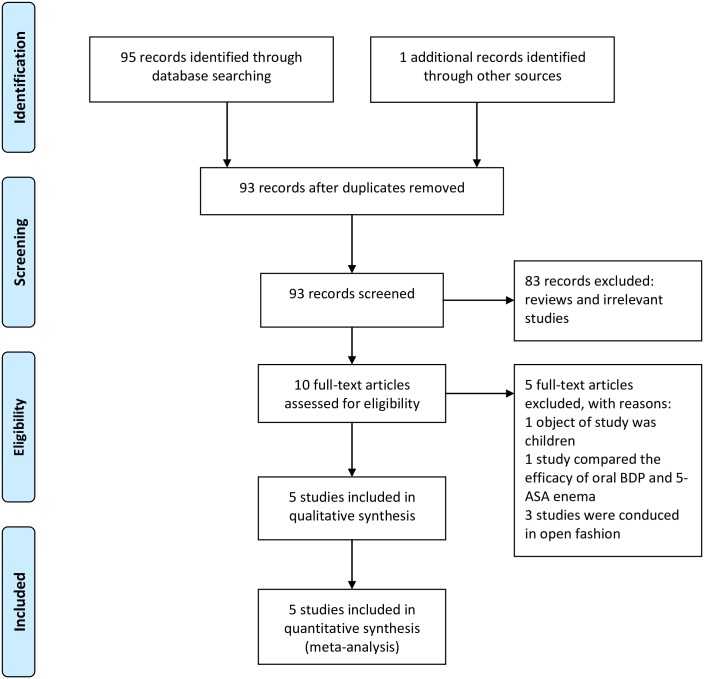
Flow diagram for selection of randomized controlled trials (RCTs) included in the meta-analysis.

A detailed study flow diagram is shown in Fig 1. The research strategy identified 95 citations through database searching and one through other sources. One article was excluded due to duplication. Of 93 records screened, 83 were excluded after examining the title and abstract. Ten studies conducted from 2001 to 2015 included data on efficacy of BDP, orally administered as tablets, in patients with active UC [24–33]. Of them, eight evaluated patients with left sided (i.e., limited to below the splenic flexure) or total colitis [24,26,28–33], one evaluated patients with left sided colitis [25], while another evaluated patients without restriction for colitis extension [28]. The sample size was precalculated only in five studies [24,30–33]. Four studies were conducted in double blind [24,30,32–33], two in single blind [25,31], and four in open fashion [26–29]. Randomization of patients was made in seven studies [24–25,28,30–33]. One study compared the efficacy of oral BDP and 5-ASA enema [25]. Five full-text articles satisfied the inclusion requirements.

The efficacy of oral BDP vs. oral 5 mg/day active controls in adult UC patients has been addressed in 5 clinical trials [24,30–33] (Table 1). One study has been designed to compare oral BDP vs. oral PD [24]. In three studies oral 5-ASA was used in control groups [31–33]. In one study with a placebo arm [30] a dose of oral 5-ASA was also allowed especially because ethical committees were reluctant to countenance a placebo arm. Therefore, the trial was in fact BDP vs. 5-ASA. In the BDP arm 5-ASA was allowed as concomitant therapy only in three RCTs [24,30,32]. Allocation concealment was adequate in these five studies [24,30–33]. Double blinding was used in four studies [24,30,32–33], with single blinding in one [31]. Agreement between investigators for trial eligibility was perfect (Kappa statistics = 1.0). The main characteristics of the selected studies are reported in Table 1.

**Table 1:** Characteristics of randomized controlled studies comparing oral beclomethasone dipropionate to oral aminosalicylates.

Year of publication	Author (country)	Number of participants	Number of patients in the treatment arms	UC characteristics	Medication (dose)	Time and evaluation parameters	Number of responders (%), ITT data	Results/conclusions
2001	Rizzello et al. [33] (Italy)	57	Group A = 19; Group B = 19; Group C = 19	Extensive or left-sided UC	Group A = BDP tab (5 mg/day) + one placebo tab; Group B = BDP tab (10 mg/day); Group C = 5-ASA tab (1.6 g/day)	4 weeks; Clin + E (DAI score)	Group A = 9 (47.4); Group B = 9 (47.4); Group C = 7 (36.8)	Similar efficacy in Clin + E for BDP 5 mg or 10 mg
2002	Rizzello et al. [32] (Italy)	119	Group A = 58; Group B = 61	Extensive or left-sided UC	Group A = BDP tab (5 mg/day) + 5-ASA tab (3.2 g/day); Group B = one placebo tab + 5-ASA tab (3.2 g/day)	4 weeks; Clin + E (DAI score) + H	Group A: remission = 34 (58.6), improvement = 10 (17.2). Group B: remission = 21 (34.4), improvement = 10 (16.4)	More efficacy in Clin + E for BDP
2003	Campieri et al. [31] (Italy)	177 (ITT analysis for efficacy = 153)	Group A = 73; Group B = 80	Extensive or left-sided UC	Group A = BDP tab (5 mg/day); Group B = 5-ASA tab (2.4 g/day)	4 weeks; Clin + E (DAI score) + H	Group A: remission = 46 (63), improvement = 11 (15.1); Group B: remission = 50 (62.5), improvement = 9 (11.3)	Similar efficacy in Clin + E + H
2003	Chiesi Farmaceutici S.p.A. Data on file [30] (France)	253	Group A = 64; Group B = 65; Group C = 66; Group D = 58	Extensive UC (>50 cm from the anal verge)	Group A = BDP tab (5 mg/day) + one placebo tab in the morning 1st day, and two placebo tab in the morning 2nd day; Group B = BDP tab (5 mg/day) + one placebo tab in the morning every day; Group C = BDP tab (10 mg/day) in the morning every day; Group D = two placebo tab in the morning every day. Allowed oral 5-ASA max 3 g/day in all groups	4 weeks; Clin + E (DAI score) + CAI score	Group A: remission 10 (15.6), improvement 41 (64.1). Group B: remission 11 (16.9), improvement 37 (56.9). Group C: remission 15 (22.7), improvement 36 (54.6). Group D: remission 7 (12.1), improvement 28 (48.3)	Similar efficacy in Clin + E between BDP groups
2015	Van Assche et al. [24] (Multinational)	282	Group A = 137; Group B = 145	Extensive or left-sided UC	Group A = BDP tab (5 mg/day) for 4 weeks followed by BDP tab (5 mg every other day) for 4 weeks; Group B = PD tab (40 mg/day for 2 weeks, and then tapered of 10 mg every 14 days during 6 weeks). Allowed oral 5-ASA max 3.2 g/day in all groups	4 weeks; Clin + E (DAI and CAI score); 8 weeks; Clin (CAI score)	Group A: remission = 25 (18.2), improvement = 59 (43.1). Group B: remission = 31 (21.4), improvement = 59 (40.7)	Similar efficacy in Clin + E for BDP and PD

5-ASA, 5-aminosalicylic acid; BDP, beclomethasone dipropionate; CAI, clinical activity index; Clin, clinical evaluation; n, number of patients; DAI, disease activity index; E, endoscopic evaluation; H, histological evaluation; PD, prednisone; tab, tablets; UC, ulcerative colitis.

The multicentre double blind trial by Rizzello et al. [33] enrolled patients not under treatment with CSs or 5-ASA compounds in the month prior to the study entry in three treatment arms with a dose-finding purpose. In the two BDP groups, oral and rectal 5-ASA were not allowed during the study period. For meta-analysis we used data of oral BDP 5 mg o.d. and oral 5-ASA. Sample size calculation was based on the hypothesis that 85% of patients treated with the higher BDP dose and 45% treated with the lower dose would achieve remission or significant clinical and endoscopic improvement at the end of the study. DAI was used to assess efficacy, and patients with DAI score reduced by at least three points from baseline were classified as responders. No significant difference in clinical changes was apparent among the three groups. Two (10.5%) steroid related AEs occurred only in the 10 mg o.d. group.

The other multicentre trial by Rizzello et al. [32] enrolled patients not under treatment with CSs in the month prior to study entry or 5-ASA at a dose >3.2g/day or sulfasalazine >2g/day for two weeks prior to study entry in two treatment arms. The study was conducted according to a randomized, double-blind, placebo-controlled design. The sample size was calculated on the assumption that 65% of patients would respond to BDP treatment and 40% would respond to placebo + 5-ASA and when a two-tailed test was employed with α = 0.05 and 1-β = 0.80. The DAI score was calculated at baseline and after 4 weeks of treatment to assess the efficacy of this therapy on UC symptoms. Patients were classified as responders if their DAI score was reduced by at least three points compared to baseline and in clinical remission with a DAI score < 3 at the end of the study. The combination of oral BDP with 5-ASA proved to be significantly more effective than 5-ASA alone (P = 0.021 for patients in clinical remission). Two out of 58 (3.4%) patients in the BDP group and 4 out of 61 (6.5%) in the placebo + 5-ASA group experienced AEs.

The multicentre trial by Campieri et al. [31] enrolled 177 patients not under treatment with CSs or 5-ASA compounds for at least one month prior to enrolment in two treatment arms. The study was performed with a multicentre, single blind, randomised and controlled design. The third-part blind observer method was used to assess efficacy. The sample size was calculated assuming that after treatment 80% of patients in the BDP group would be in remission compared with 60% in the 5-ASA group, with alpha error set at 0.05 and statistical power at 80%. The DAI score was calculated at baseline and after 4 weeks of treatment to establish the UC treatment efficacy. Patients were classified in clinical remission after a 4-week treatment with a DAI score <3. The clinical improvement was defined as a reduction of at least three points in the DAI score from baseline values (responders). Moreover, patients were classified in clinical remission with a DAI score < 3 at the end of the study. Efficacy and safety ITT analyses were performed on all patients who had received at least one dose of study medication and who had attended at least one visit after baseline. According to the ITT analysis, the efficacy variable DAI was evaluated in 73 patients in the BDP group and 80 patients in the 5-ASA group. The percentage of patients in clinical remission and with a significant clinical improvement did not differ between the two treatment groups. One patient in the BDP group and one patient in the 5-ASA group experienced AEs.

We also considered the regulatory placebo-controlled trial entitled ‘Multicentre double blind randomised, balanced parallel group, controlled, dose range finding study of 5 mg every 2 days, 5 mg/day, and 10 mg/day of Beclomethasone Dipropionate enteric coated tablets versus placebo in mild to moderate extensive ulcerative colitis patients relapsing under oral aminosalicylates (like mesalazine, sulfasalazine) preventive therapy’ [30]. It is a study (Number RA/PR/1405/001/00) whose final report was closed on September 19^th^ 2003 and used for registration purposes. The protocol was approved by competent local Regulatory Committee (France) and different Ethics Committees. The study was sponsored by Chiesi S.A., 11 Avenue Dubonnet, 92400 Courbevoie, France, and enrolled patients in four treatment arms. In all patients oral aminosalicylates had to be in stable regimen for 6 weeks at least at the inclusion in the study at a maximum dosage of 3 g/day and maintained at the same dose during all the study. Rectal treatment had to be stopped 15 days before the inclusion. The study duration was 4 weeks. For the meta-analysis we used data of Group B and D. Randomization was centralised. For each group, the sample size of 65 patients was calculated to detect a 20% absolute difference in the proportion of patients entering remission while taking the less effective of the three doses evaluated, and assuming a response rate to placebo of 5%, with significance (alpha) set at 0.050 and a power of 90%. Estimating a dropout rate of 10%, a maximum number of 290 patients were to be enrolled in order to obtain the 260 completed cases. DAI and CAI were used to assess drug efficacy. Patients were classified in clinical remission after a 4-week treatment with a DAI score ≤ 1 and an endoscopically documented mucosal healing. The responder rate was defined by at least one of following criteria: Differential DAI scores between Visit 4 and Visit 2 ≥3; Differential endoscopic score between Visit 4 and Visit 2 ≥1; Differential CAI score between Visit 4 and Visit 2 ≥3. Twenty-four patients (9.5%) prematurely discontinued study, with 5 of them having 21-day maximum exposure to study treatment at their final visit evaluation. The differences between groups without adjustment were not statistically significant regarding remission and responder rates. A total number of 56 AEs were reported during the study: 13 (in 11 patients) in the placebo group, 14 (in 10 patients) in the 5 mg every two days group, 12 (in 7 patients) in the 5 mg group and 17 (in 13 patients) in the 10 mg group.

The multicentre, multinational trial by Van Assche et al. [24] enrolled patients that were randomized in two treatment arms. The use of rectal therapies or non-steroidal anti-inflammatory drugs within 14 days, systemic oral or parenteral corticosteroids within 30 days, immunosuppressors in the previous 3 months, and anti-tumour necrosis factor-α therapy within 6 months from the screening visit and throughout the study were not allowed. Oral aminosalicylate drugs were allowed at a stable regimen for at least 14 days before study entry at a maximum dose of 3.2 g/day for mesalazine, 6.75 g/day for balsalazide, 2 g/day for olsalazine, and 3 g/day for sulfalsalazine. The sample size was calculated in order to reject the null hypothesis that the investigational treatment was inferior to the reference treatment, using a two-sided 95% confidence interval. Primary efficacy and safety end points were assessed after 4 weeks of treatment and data were used for this meta-analysis. The primary efficacy end point was to demonstrate the non-inferiority of oral BDP compared with PD in terms of the DAI score. Clinical response was defined as a DAI score <3 or a reduction of the DAI score by at least 3 points for patients with a baseline DAI from 7 to 9. The primary safety variable was to demonstrate a better safety profile in terms of patients who experienced AEs specifically associated by the investigator to corticosteroid treatment and related to the study drug (steroid-related AEs) and reduction of endogenous cortisol production below 150 nmol/l. Secondary end points were also calculated after 4 and 8 weeks of treatment. Efficacy and safety ITT analyses were performed on all patients who had received at least one dose of study medication and who had attended at least one visit after baseline. The percentage of patients in clinical remission and with a significant clinical improvement did not differ between the two treatment groups. Fifty-three patients in the BDP group and 68 in the PD group experienced AEs without a difference between the two groups.

### Risk of bias

Rizzello et al. study [33]: random sequence generation method was clearly specified. The randomization list was made by the Biometrics Section of Chiesi Farmaceutici S.p.A. Cards in sealed opaque envelopes were used for allocation concealment to the three study groups. Double blinding was assured. Outcome data were reported for all enrolled patients. Outcomes of interest included in the study protocol were completely reported. In all cases the missing data were related to the true outcome. Clear information was provided on concomitant treatments.

Rizzello et al. study [32]: random sequence generation was clearly specified. Treatment allocation was made from blocks of four numbers produced by a computer-generated randomization list. Cards in sealed opaque envelopes were used for allocation concealment to the two study groups. Double blinding was assured. Outcome data were reported for all enrolled patients. Outcomes of interest included in the study protocol were completely reported. In all cases the missing data were related to the true outcome. Clear information was provided on concomitant treatments.

Campieri et al. study [31]: random sequence generation was clearly specified. Treatment allocation was made from blocks of four numbers produced by a computer-generated randomization list. Cards in sealed opaque envelopes were used for allocation concealment to the two study groups. Single blinding was assured. The third-part blind observer method was used to assess the efficacy of the test treatments. To avoid biased efficacy assessments of UC, the investigators who performed endoscopic and histological examinations, and the evaluation of the clinical symptoms were blinded to patient’s treatment assignment, whereas the investigators in charge of treatment allocation were excluded from all efficacy assessments. Outcome data were reported for all enrolled patients and all withdrawals were related to the true outcome. Not all withdrawal patients were included in the ITT analysis because of the prespecified ITT criteria. Outcomes of interest included in the study protocol were completely reported. Clear information was provided on concomitant treatment.

Chiesi S.A. data on file [30]: random sequence generation method was clearly specified. Randomization was provided by Chiesi S.A. At the beginning of the study all test treatments were sent to a central pharmacist for dispatching. After the screening visit of one patient, each investigator faxed the prescription to the central pharmacist who sent the test treatment according to the randomization list. In the establishments where a pharmacist was available the test treatment was sent to the pharmacist. In the establishments where a pharmacist was not available the test treatment was sent to the investigator. Cards in sealed opaque envelopes were used for allocation concealment to the four study groups. Double blinding was assured. Outcome data were reported for all enrolled patients. Outcomes of interest included in the study protocol were completely reported. In most of cases the missing data were related to the true outcome, except in two cases where the discontinuation was related to patients’ personal reasons. Clear information was provided on concomitant treatment.

Van Assche et al. study [24]: random sequence generation was clearly specified. Treatment allocation was made according to a balanced-block design (1:1 randomization scheme) by Chiesi Farmaceutici S.p.A. The patient was identified with a randomization number, and the investigator assigned the treatment starting from the lowest available randomization number at the site. Individual subject treatment code sealed in opaque envelopes were provided to the investigators reporting the test treatment and randomization numbers for emergency situations. Double blinding was assured. Outcome data were reported for all enrolled patients. Outcomes of interest included in the study protocol were completely reported. In all cases the missing data were related to the true outcome. Clear information was provided on concomitant treatment.

**Fig 2:**
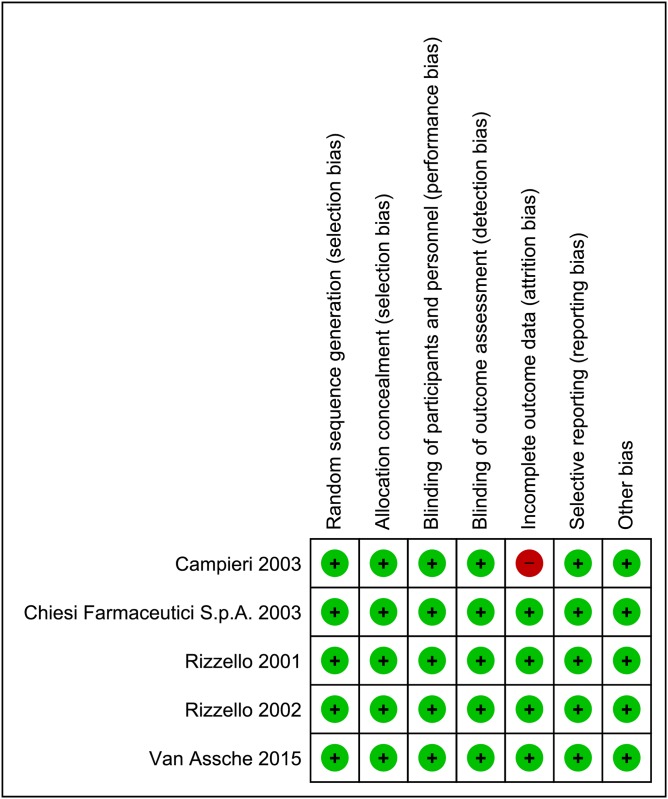
Risk of bias summary. **This risk of bias tool incorporates the assessment of randomization (sequence generation and allocation concealment), blinding (participants and outcome assessors), incomplete outcome data, selective outcome reporting and other risk of bias.** The items were judged as ‘low risk’ ‘unclear risk’ or ‘high risk’. Green means ‘low risk’ and red means ‘high risk’.

The methodological quality assessment of the 5 included studies is presented in Fig 2. We judged the risk of selection bias as low in all controlled studies. In all cases a blinding method was adopted. Attrition bias and reporting bias, as well as other sources of bias were judged as low for all controlled studies except for Campieri et al. study [31] because of the imbalanced missing data (18.9% vs. 8.1%).

### Detail of efficacy and safety analysis

Efficacy and safety of oral BDP vs. oral PD or 5-ASA has been addressed in 5 RCTs and data are summarized in Table 2. According to the ITT analysis 369 UC patients were treated with BDP and 370 with PD or 5-ASA. After 4 weeks of treatment 242 (65.6%) BDP patients and 222 (60.0%) PD or 5-ASA patients were considered responders. Data obtained from 4 RCTs [23,30–32] indicated that 116 (31.4%) patients in the BDP group and 109 (29.5%) in the PD or 5-ASA group were in remission after 4 weeks of treatment. In the BDP group and in the PD or 5-ASA group 63 (17.1%) and 84 (22.7%) patients experienced AEs, respectively. In one study AEs were not observed in the two groups [33] and the OR was not estimable. Funnel plots for each of these study sets are included in S1 Fig.

**Table 2:** Data for the efficacy and safety analysis.

Author	BDP 5mg/day, n (%)						PD or 5-ASA, n (%)					
	Number of pts. for ITT analysis	Responders	Remission	Improvement	Treatment failure	Pt. with AEs	Number of pts. for ITT analysis	Responders	Remission	Improvement	Treatment failure	Pt. with AEs
Rizzello et al. [33]	19	9 (47.4)	-	-	10 (52.6)	0 (0)	19	7 (36.8)	-	-	12 (63.2)	0 (0)
Rizzello et al. [32]	58	44 (75.9)	34 (58.6)	10 (17.2)	14 (24.1)	2 (3.5)	61	31 (50.8)	21 (34.4)	10 (16.4)	30 (49.2)	4 (6.6)
Campieri et al. [31]	73	57 (63.3)	46 (63)	11 (15.1)	16 (17.8)	1 (1.1)	80	59 (73.8)	50 (62.5)	9 (11.3)	21 (26.3)	1 (1.2)
Chiesi Farmaceutici, data on file. [30]	65	48 (73.9)	11 (16.9)	37 (56.9)	17 (26.2)	7 (10.8)	58	35 (60.4)	7 (12.1)	28 (48.3)	23 (39.7)	11 (19.0)
*Van Assche et al. [24]	137	84 (61.3)	25 (18.2)	59 (43.1)	53 (38.7)	53 (38.7)	145	90 (62.1)	31 (21.4)	59 (40.7)	55 (37.9)	68 (46.9)

5-ASA, 5-aminosalicylic acid; AEs, adverse events; BDP, beclomethasone dipropionate; PD, prednisone. Data are number of patients (%).

*According to the ITT analysis as specified by authors, the primary efficacy and safety endpoints were evaluated after 4 weeks of treatment.

#### Clinical response

**Fig 3:**
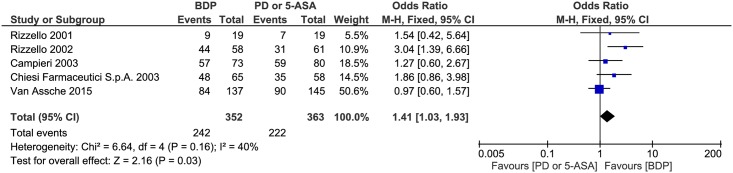
Forest plot of randomized controlled trials of oral BDP vs. oral PD or 5-ASA in inducing clinical response in ulcerative colitis. BDP, beclomethasone dipropionate. PD, prednisone. 5-ASA, mesalazine. M-H, Mantel-Haenszel.

There was moderate heterogeneity between studies (P = 0.16, I^2^ = 40%), so we used the fixed-effects model to pool the data. The overall estimate indicated that the pooled OR was 1.41 (95% CI = 1.03–1.93, P = 0.03) (Fig 3). Given that the lower and upper confidence limits for the OR exceeds 1.0 and that the horizontal block lies to the right of the vertical line, it indicates that the clinical response of BDP is better than PD or 5-ASA.

#### Clinical remission

**Fig 4:**
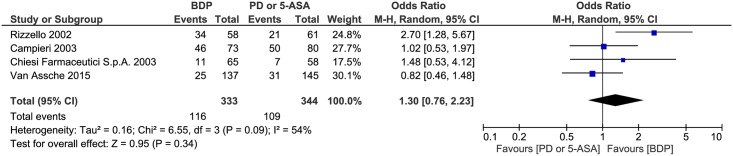
Forest plot of randomized controlled trials of oral BDP vs. oral PD or 5-ASA in inducing clinical remission in ulcerative colitis. BDP, beclomethasone dipropionate. PD, prednisone. 5-ASA, mesalazine. M-H, Mantel-Haenszel.

A heterogeneity test revealed significant heterogeneity among the studies (P = 0.09, I^2^ = 54%), so the random-effects model was used. A pooled analysis revealed that there was no significant difference between the BDP and PD or 5-ASA groups (OR 1.30, 95% CI = 0.76–2.23, P = 0.34) (Fig 4).

#### Safety

**Fig 5:**
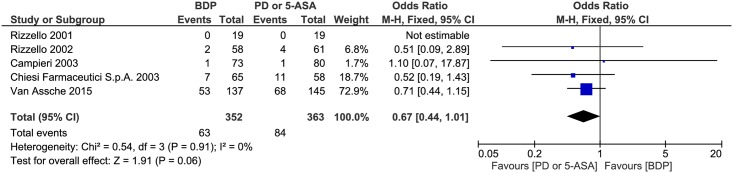
Forest plot of randomized controlled trials of oral BDP vs. oral PD or 5-ASA on adverse events appearance in ulcerative colitis. BDP, beclomethasone dipropionate. PD, prednisone. 5-ASA, mesalazine. M-H, Mantel-Haenszel.

No statistically significant heterogeneity was detected for this comparison (P = 0.91, I^2^ = 0%). A pooled analysis revealed that there was no significant difference between the BDP and PD or 5-ASA groups (OR 0.67, 95% CI = 0.44–1.01, P = 0.06) (Fig 5). Given that the upper confidence limit for the OR is 1.01 and that the p-value is 0.06 for this outcome as well as that the horizontal block lies to the left of the vertical line, this result suggests a more favourable safety profile of BDP compared to 5-ASA and PD.

### Subgroup analysis

We performed a subgroup analysis to determine efficacy and safety of oral BDP vs. oral 5-ASA. According to the ITT analysis, a total of 215 UC patients was treated with BDP and 218 with 5-ASA. After 4 weeks of treatment 158 (73.5%) BDP patients and 132 (60.6%) 5-ASA patients were considered responders. Data obtained from 3 RCTs [30–32] indicated that 91 (46.4%) patients in the BDP group and 78 (39.2%) in the 5-ASA group were in remission after 4 weeks of treatment. Moreover, in the BDP group and in the 5-ASA group 10 (4.7%) and 16 (7.3%) patients experienced AEs, respectively. Funnel plots for each of these study sets are included in S1 Fig.

#### Clinical response

**Fig 6:**
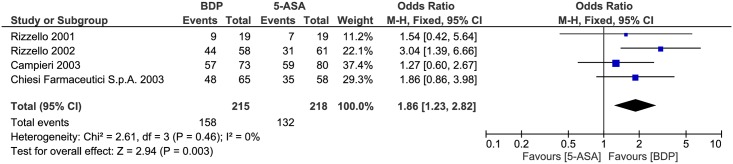
Forest plot of randomized controlled trials of oral BDP vs. oral 5-ASA in inducing clinical response in ulcerative colitis. BDP, beclomethasone dipropionate. 5-ASA, mesalazine. M-H, Mantel-Haenszel.

Clinical response was compared between the two drug treatments. No statistically significant heterogeneity was detected for this comparison (P = 0.46, I^2^ = 0%). A pooled analysis revealed that the clinical response of BDP is better than 5-ASA (OR 1.86, 95% CI = 1.23–2.82, P = 0.003) (Fig 6).

#### Clinical remission

**Fig 7:**
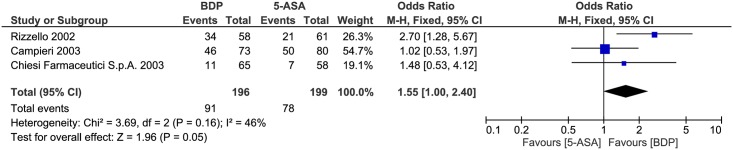
Forest plot of randomized controlled trials of oral BDP vs. oral 5-ASA in inducing clinical remission in ulcerative colitis. BDP, beclomethasone dipropionate. 5-ASA, mesalazine. M-H, Mantel-Haenszel.

Clinical remission was compared between the two drug treatments. There was moderate heterogeneity between studies (P = 0.16, I^2^ = 46%), so we used the fixed-effects model to pool the data. The overall estimate indicated that the pooled OR was 1.55 (95% CI = 1.00–2.40, P = 0.05) and there was no obvious difference between the two groups (Fig 7). Given that the lower confidence limit for the OR is 1.0 and that the p-value is 0.05 for this outcome as well as that the horizontal block lies to the right of the vertical line, it indicates that the potential clinical efficacy of BDP may be better than 5-ASA.

#### Safety

**Fig 8:**
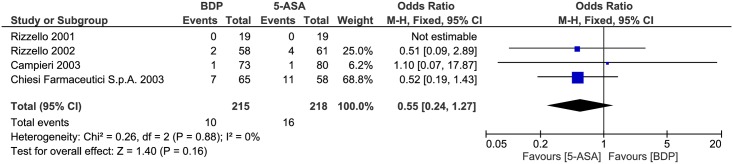
Forest plot of randomized controlled trials of oral BDP vs. oral 5-ASA on adverse events appearance in ulcerative colitis. BDP, beclomethasone dipropionate. 5-ASA, mesalazine. M-H, Mantel-Haenszel.

Safety was compared between the two drug treatments. No statistically significant heterogeneity was detected for this comparison (P = 0.88, I^2^ = 0%). A pooled analysis revealed that there was no significant difference between the BDP and 5-ASA groups (OR 0.55, 95% CI = 0.24–1.27, P = 0.16) (Fig 8).

## Discussion

Right now, few RCTs have been produced to evaluate efficacy and safety of oral prolonged release BDP against active oral comparators. A recent comparative study between oral prolonged release BDP 5 mg and oral PD 40 mg in extensive or left-sided mild to moderate UC showed that both drugs achieved similar clinical and endoscopic efficacy [24]. Four controlled studies, conducted between 2001 and 2003, established efficacy and safety of oral controlled release BDP 5 mg vs. oral 5-ASA [30–33]. In particular, a significant difference in clinical remission was found only in the study of Rizzello et al. [32], demonstrating that the combination of oral BDP with 5-ASA is more effective than 5-ASA alone. The other three studies, two of them planned with a dose-finding aim [30,33], did not evidenced differences between the two treatment arms, because they were not designed for this purpose. This meta-analysis shows that the treatment with oral BDP 5 mg/day induces a significant better clinical response compared to oral 5-ASA. The effect is detectable even when the comparison to PD is included in the meta-analysis. Data on remission after only 4-week treatment period indicates that the potential clinical efficacy of BDP may be better than 5-ASA. These results are not surprising and may be explained by the higher surface-active anti-inflammatory properties of BDP [7,39] than 5-ASA. Obviously, this difference is lost when the comparison with PD is added in the meta-analysis for the strong anti-inflammatory properties of this systemic first generation CS. The safety analysis showed no differences between BDP and 5-ASA. The lack of difference is maintained even when the study with PD is included (OR 0.67, 95% CI = 0.44–1.01, P = 0.06). However, the trend of difference is clear and indicates a more favourable safety profile of BDP compared to 5-ASA and PD. This non-significance may be due to the limited number of studies included in the meta-analysis. Moreover, it is of note that the search for differences in AEs, including reduced cortisol concentration, in the Van Assche’s study [24] was influenced by the analytical method used for cortisolemia [48], conducing to a lower number of AEs observed in the PD arm. However, a large burden of data in adults affected by UC from non-controlled ‘real life’ studies [25–27,29] or in a controlled study on children [28] confirmed a good efficacy and safety profile of oral BDP administrated at different dosages (from 5 to 15 mg/day) for at least 4 weeks.

Recently a systematic review and meta-analysis about efficacy and safety of beclomethasone dipropionate (enema and oral formulations) in the treatment of ulcerative colitis was published [49]. Authors concluded that there is no difference between beclomethasone dipropionate (oral and rectal) and 5-ASA group even though the clinical efficacy of oral BDP may be better of 5-ASA after statistical deduction. In this meta-analysis only two paper with oral BDP were retrieved for this purpose. In our meta-analysis, we considered 5 RCTs after 4-week treatment period. Our results obviously are more robust and clearly indicate that oral BDP 5 mg/day induces a significant better clinical response compared to oral 5-ASA (OR 1.86, 95% CI = 1.23–2.82, P = 0.003), and the effect is detectable even when the comparison to prednisone is added (OR 1.41, 95% CI = 1.03–1.93, P = 0.03).

In Italy and in few other European and non-European countries, oral controlled-release BDP formulations have been approved for UC [1] for the treatment of mild to moderate ulcerative colitis in active phase, as add-on therapy to 5-ASA containing drugs in patients who are non-responders to 5-ASA therapy in active phase. In two recent guidelines [3,50] the exact role of oral CSs preparations with a colonic release mechanism and low systemic bioavailability, such as BDP or budesonide (MMX), with efficacy for induction of remission and fewer systemic CSs AEs, is not stated. This may be due to the scarce or contrasting literature produced until now in terms of RCTs for these second-generation steroids. It is noteworthy that while there is no direct comparison to systemic steroids for budesonide [51], the efficacy and safety of oral prolonged release BDP vs. oral PD is well established in a controlled trial [24], but data were published after guidelines.

This meta-analysis confirms the good safety profile of 5 mg/day prolonged release oral BDP. The newer steroids like BDP and budesonide exert their activity topically. Pro-drug BDP is hydrolysed to the active metabolite 17-BMP via esterase enzymes of mucosal cells where it exerts a potent anti-inflammatory effect. After mucosal absorption, BDP is subject to high first-pass hepatic metabolism and metabolized into inactive products that reach the systemic circulation. Thanks to this extensive first-pass metabolism, BDP has a limited impact on the HPA axis with fewer systemic effects than traditional CSs [52]. Interestingly, morning plasma cortisol levels seem to have a dose-dependent decrease in BDP and budesonide [30,51]. If confirmed, this does call into question whether the ‘second-generation’ of topically acting steroids have safety advantages with dosage higher than 5 mg/day compared to systemic CSs. Although the second-generation steroids may contribute to the improvement in the quality of life for patients not responsive to 5-ASA compounds, no data are available for the risk of inducing steroid-dependence, that account for up to 25% during the use of first generation steroids [1], or AEs after a longer treatment period or with the use of higher dose of these drugs.

In conclusion, available data shows that oral prolonged release BDP is more effective than 5-ASA in the treatment of mild to moderate UC to achieve clinical remission/response with low AEs at the dose of 5 mg/day. The recommended placement of second-generation corticosteroid therapies within current treatment paradigm for mild-to-moderate UC, is reserved for patients who are unresponsive or intolerant 5-ASA treatments before starting systemic steroids or thiopurines [53].

### Limitation and strength of the analysis

All trials included in our analysis have some methodological limitations. In particular, there is no effective dose standard for treatment with 5-ASA (from 1.6 g/day to 3.2 g/day), so there was a lack of uniformity of drug dosage among the various studies. Moreover, in two studies 5-ASA was not allowed as concomitant treatment in the BDP arm. Study limitations also included a small sample size in some trials. To limit the risk of publication bias, we did not impose restrictions by language or year of publication and made any attempt to identify all trials in order to obtain data that strengthened our meta-analysis. In particular, an important study used for registration purposes was obtained for the meta-analysis. Further strength comes from the fact that the same BDP dose and trial duration was used in all studies we compared.

## Supporting Information

S1 Fig Funnel plot of response, remission, and adverse events for oral BDP vs. oral PD or 5-ASA, and for oral BDP vs. oral 5-ASA alone. (PDF)

S1 File PRISMA Checklist. (DOC)

S2 File Excluded full-text articles. (DOCX)
