# Supplementary material for: Efficacy and Safety of Oral Beclomethasone Dipropionate in Ulcerative Colitis: A Systematic Review and Meta-Analysis
Source: PLoS One. 2016 Nov 15;11(11):e0166455. doi: 10.1371/journal.pone.0166455 (PMC5113024; doi:10.1371/journal.pone.0166455)
Supplement: S1 Fig — (PDF) [file pone.0166455.s001.pdf]

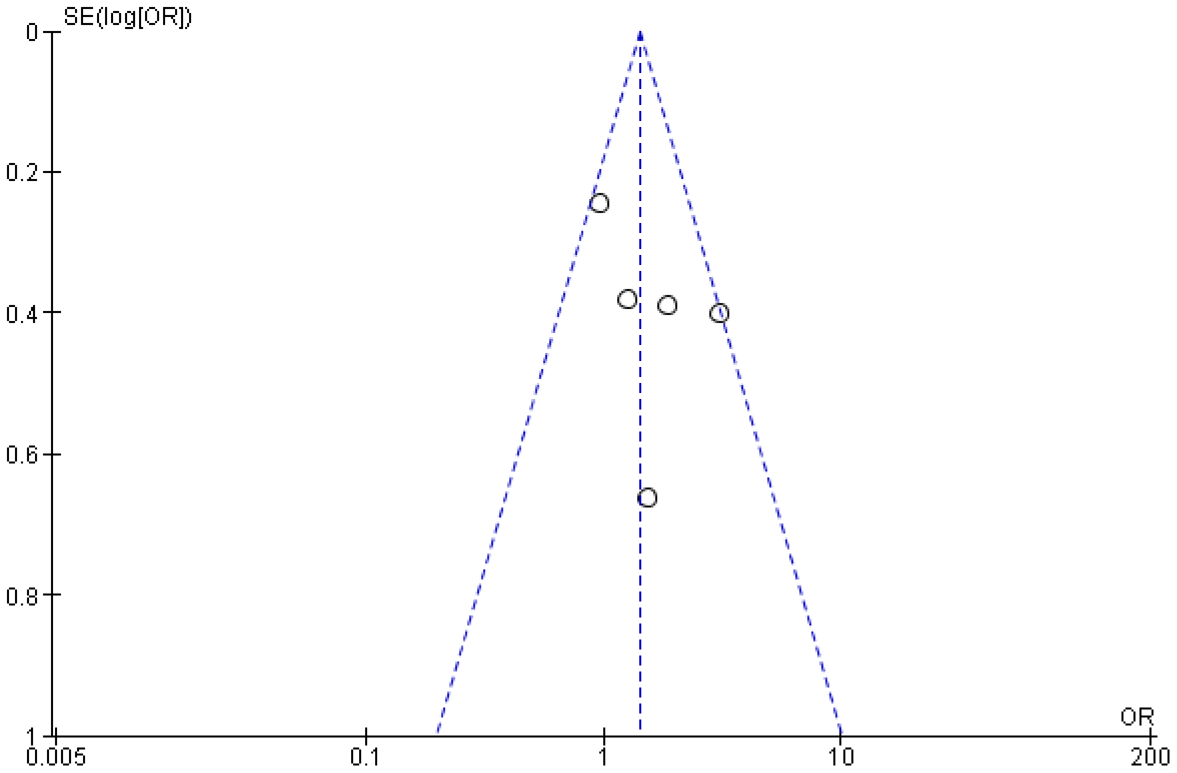

Funnel plot of comparison for oral BDP vs. oral PD or 5-ASA in inducing clinical response in ulcerative colitis.

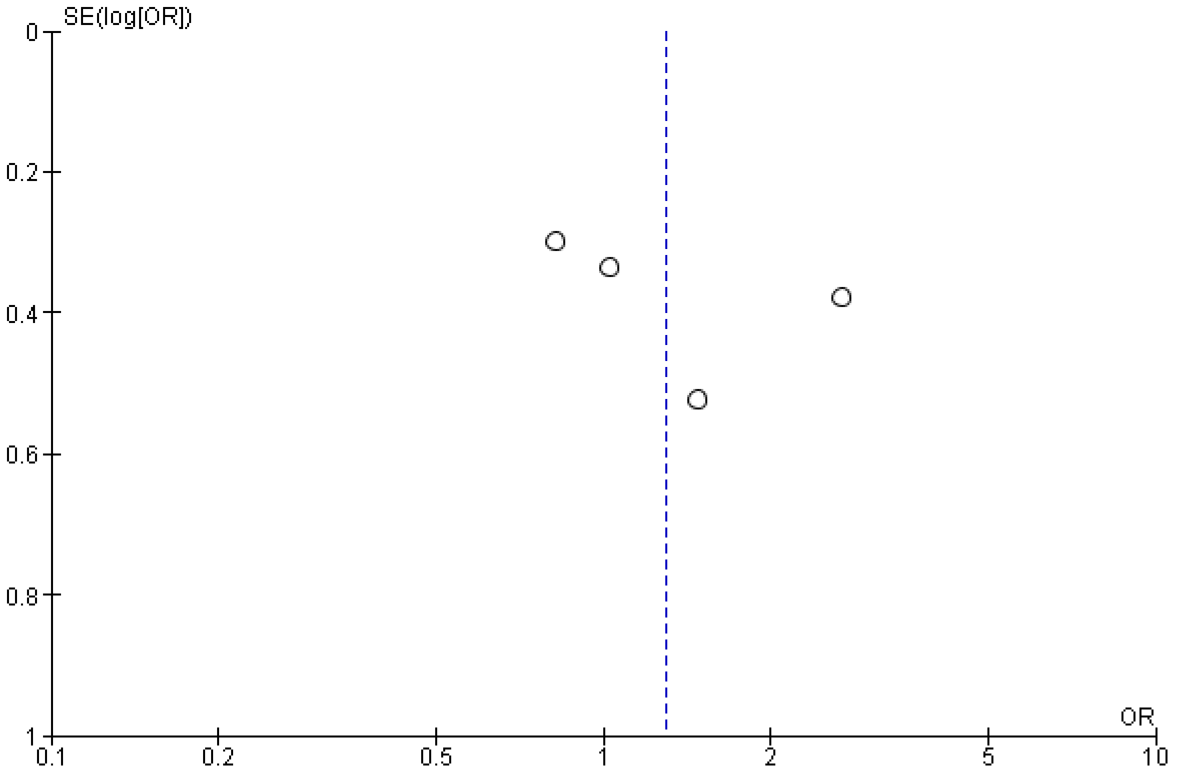

Funnel plot of comparison for oral BDP vs. oral PD or 5-ASA in inducing clinical remission in ulcerative colitis.

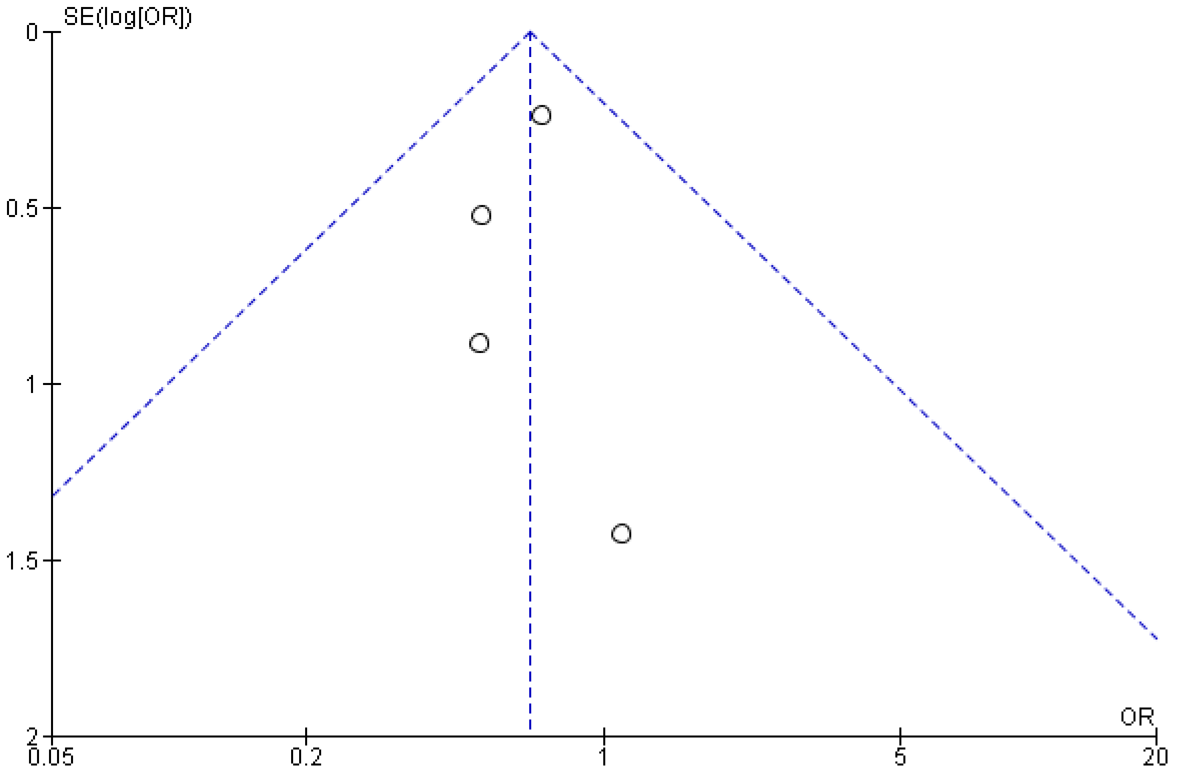

Funnel plot of comparison for oral BDP vs. oral PD or 5-ASA on adverse events appearance in ulcerative colitis.

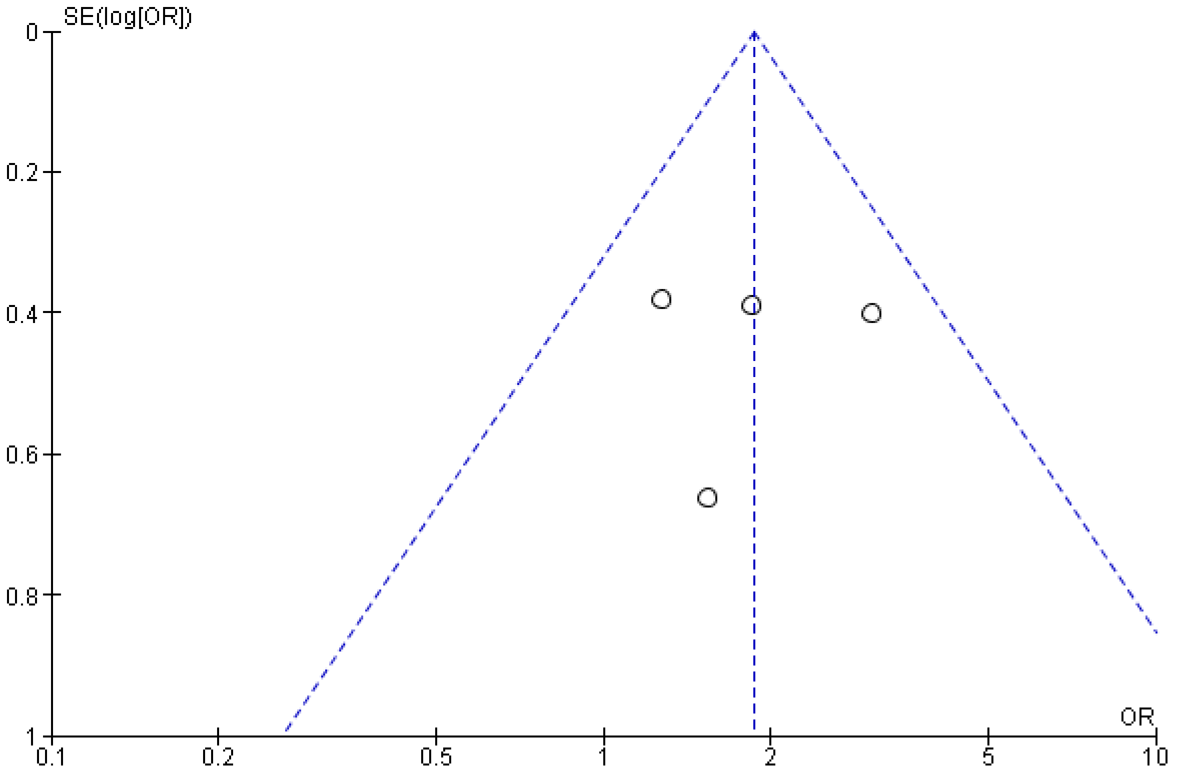

Funnel plot of comparison for oral BDP vs. oral 5-ASA in inducing clinical response in ulcerative colitis.

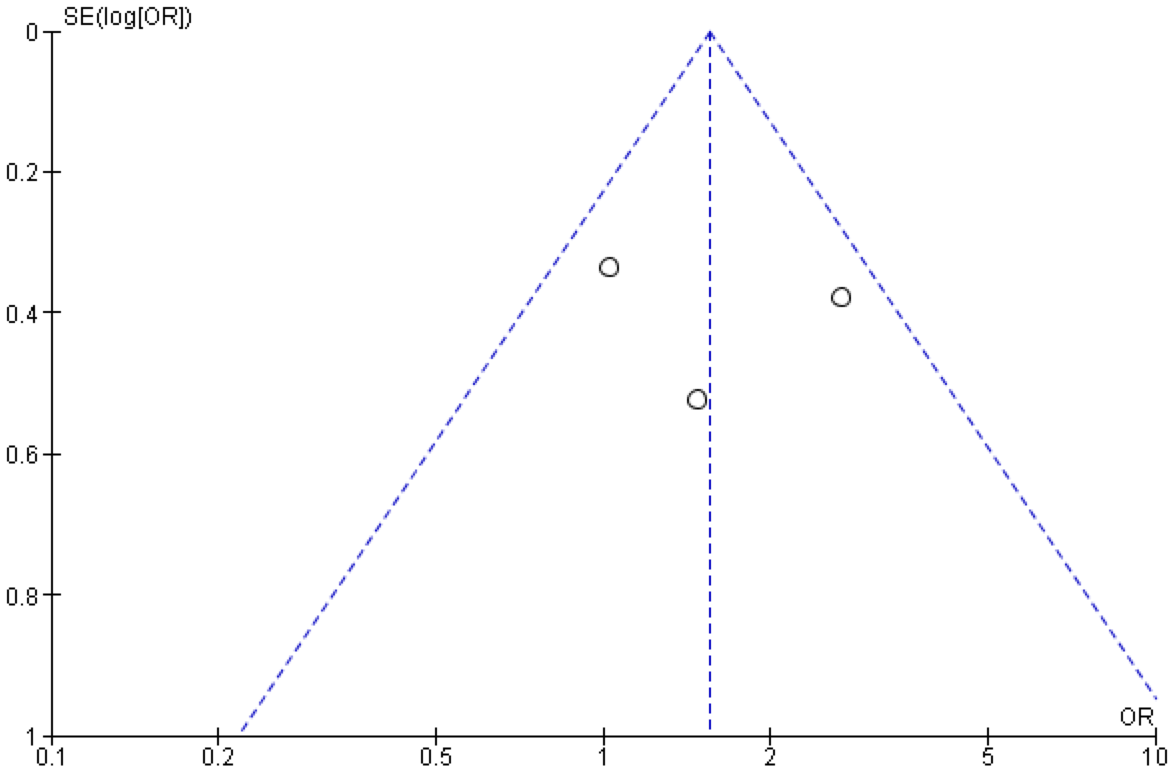

Funnel plot of comparison for oral BDP vs. oral 5-ASA in inducing clinical remission in ulcerative colitis.

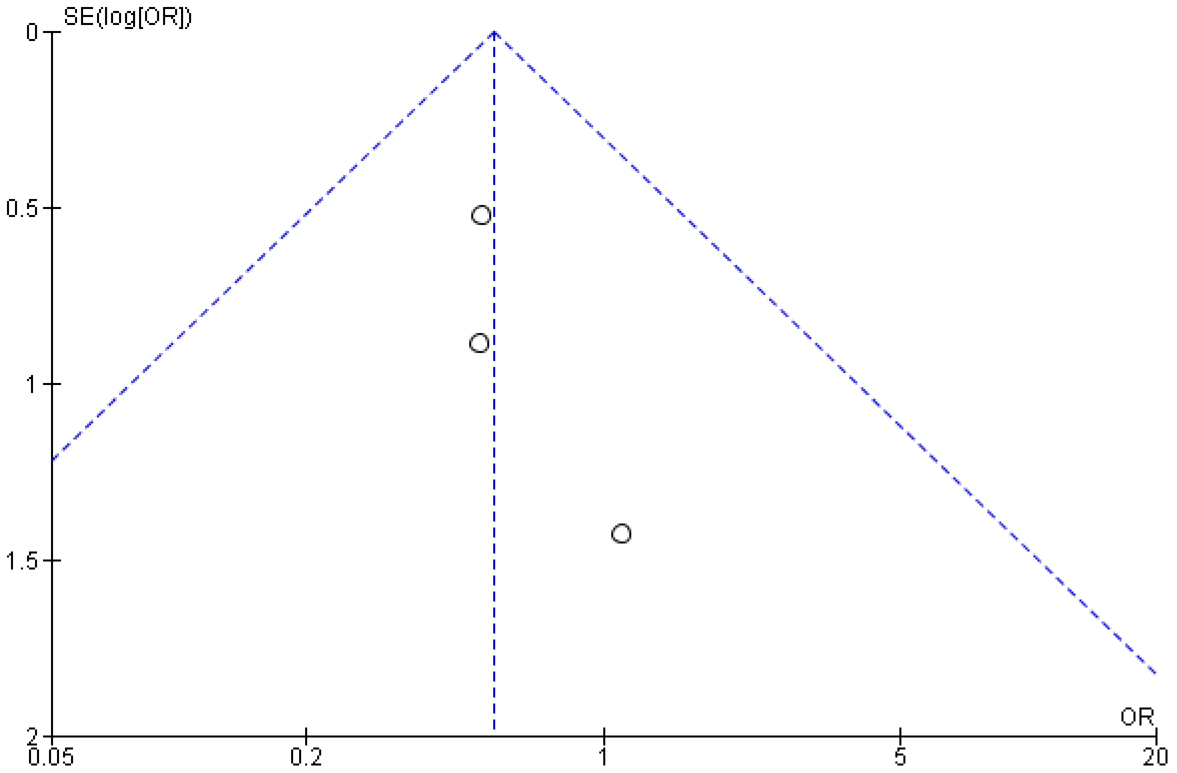

Funnel plot of comparison for oral BDP vs. oral 5-ASA on adverse events appearance in ulcerative colitis.
