# Supplementary material for: Efficacy and Safety of Oral Beclomethasone Dipropionate in Ulcerative Colitis: A Systematic Review and Meta-Analysis
Source: PLoS One. 2016 Nov 15;11(11):e0166455. doi: 10.1371/journal.pone.0166455 (PMC5113024; doi:10.1371/journal.pone.0166455)
Supplement: S2 File — (DOCX) [file pone.0166455.s003.docx]

Excluded full-text articles.

Four studies were excluded because they were planned in open fashion.

Di Caro S, Raimondo D, Mangiavillano B, Masci E, Mariani A, Gasbarrini A, et al. Oral high dose Beclomethasone dipropionate for treatment of active ulcerative colitis. Gastroenterology Insights 2012; 4: e9. [DOI: 10.4081/gi.2012.e9]

Nunes T, Barreiro-de Acosta M, Nos P, Marin-Jiménez I, Bermejo F, Ceballos D, et al. Usefulness of oral beclometasone dipropionate in the treatment of active ulcerative colitis in clinical practice: the RECLICU Study. J Crohns Colitis 2010; 4: 629-636 [PMID: 21122572 doi: 10.1016/j.crohns.2010.07.003]

Romano C, Famiani A, Comito D, Rossi P, Raffa V, Fries W. Oral beclomethasone dipropionate in pediatric active ulcerative colitis: a comparison trial with mesalazine. J Pediatr Gastroenterol Nutr 2010; 50: 385-389 [PMID: 20179636 doi: 10.1097/MPG.0b013e3181bb3457]

Papi C, Aratari A, Moretti A, Mangone M, Margagnoni G, Koch M, et al. Oral beclomethasone dipropionate as an alternative to systemic steroids in mild to moderate ulcerative colitis not responding to aminosalicylates. Dig Dis Sci 2010; 55: 2002-2007 [PMID: 19937467 doi: 10.1007/s10620-009-0962-6]

One study was excluded because compared oral BDP 5mg/day and 5-ASA enema

Pica R, Unim H, Cassieri C, Avalllone Ev, Zippi M, Paoluzi P. Oral beclomethasone dipropionate vs 5-ASA enema in active UC: Lower efficacy but better compliance. J Gastroenterol Hepatol 2013; 28 (Suppl. 3): 577 [DOI: 10.1111/jgh.12363_2]
